# Application of Molecularly Imprinted Polymers to Selective Removal of Clofibric Acid from Water

**DOI:** 10.1371/journal.pone.0078167

**Published:** 2013-10-31

**Authors:** Chaomeng Dai, Juan Zhang, Yalei Zhang, Xuefei Zhou, Shuguang Liu

**Affiliations:** 1 College of Civil Engineering, Tongji University, Shanghai, China; 2 State Key Laboratory of Pollution Control and Resource Reuse, Tongji University, Shanghai, China; 3 UNEP-Tongji Institute of Environment for Sustainable Development, Tongji University, Shanghai, China; University of Sassari, Italy

## Abstract

A new molecularly imprinted polymer (MIP) adsorbent for clofibric acid (CA) was prepared by a non-covalent protocol. Characterization of the obtained MIP was achieved by scanning electron microscopy (SEM) and nitrogen sorption. Sorption experimental results showed that the MIP had excellent binding affinity for CA and the adsorption of CA by MIP was well described by pseudo-second-order model. Scatchard plot analysis revealed that two classes of binding sites were formed in the MIP with dissociation constants of 7.52±0.46 mg L^−1^ and 114±4.2 mg L^−1^, respectively. The selectivity of MIP demonstrated higher affinity for CA over competitive compound than that of non-imprinted polymers (NIP). The MIP synthesized was used to remove CA from spiked surface water and exhibited significant binding affinity towards CA in the presence of total dissolved solids (TDS). In addition, MIP reusability was demonstrated for at least 12 repeated cycles without significant loss in performance.

## Introduction

The presence of human pharmaceuticals and their metabolites in the aquatic environment has drawn significant attention during the past decade, due to their potential in altering the normal endocrine function and physiological status of animals and humans [1], [2]. Among these compounds, clofibric acid (CA) is increasing concerndue to its frequent detection in treated water, surface water, groundwater and drinking water [3]–[5]. CA is the metabolite and active principle of the blood lipid regulators clofibrate, etofyllin clofibrate, and etofibrate, and is also considered a potential endocrine disruptor, since it interferes with the synthesis of cholesterol [3]. Nowadays, this compound is regarded as one of the most persistent drug residues with an estimated persistence in the environment of 21 years, being frequently detected in environment monitoring of pharmaceuticals all around the world [1]. Ternes measured up to 1.6 µg L^−1^ of CA in the effluent of a German treatment plant [6]. The concentration of CA was up to 103 ng L^−1^ in the Detroit River at the inlet of a drinking treatment plant [7]. It was also reported in samples from the Mississippi River and Lake Pontchartrain, Louisiana at concentrations range from 6–10 ng L^−1^ [8]. Recent findings of CA in environmental water samples in China stress the fact that its occurrence is part of a global phenomenon [9]. The potential long-term effects of CA are not yet known, however, they should not be underestimated due to the similarity between its structure and those of some phenoxyacid herbicides such as Mecoprop [10].

Previous investigations have demonstrated that biodegradation of CA in wastewater treatment plants (WWTPs) was limited [11]. For example, Zorita et al. reported 55% CA removal in a conventional WWTP in Sweden and it could be improved to 61% in tertiary treatment process by additional chemical treatment following a sand filter [12]. However, Zwiener & Frimmel investigated the biodegradation of CA in short-term tests with biofilm reactor and found only 5% of CA could be eliminated [13]. Advanced oxidation processes (AOPs) and adsorption are potential treatment processes that might improve the removal efficiency of CA in municipal wastewater treatment plants. However, CA was poorly degraded by AOPs such as ozonolysis [14], [15], H_2_O_2_/UV, sunlight and UV photolysis and TiO_2_/UV [16]. Also, economic analysis indicated that wastewater treatment with advanced techniques may be economically and environmentally undesirable due to the increased energy consumption and associated economic costs, as well as CO_2_ emission [17]. Adsorbents based on activated carbon are commonly used in advanced wastewater treatment for the removal of organic contaminants due to the availability and lower cost of activated carbon compared to other novel adsorbents. However, activated carbon is difficult to regenerate, requiring high pressure and/or temperature, and also tends to saturate [17]. Moreover, these methods are normally non-selective. Hence, the necessity for developing a selective, simple, and reliable water treatment process is becoming attractive.

Nowadays, molecular imprinting is a useful technique to construct specific sites for the target compounds in the preparation of molecularly imprinted polymer (MIP), which can effectively recognize specific adsorbate during the sorption process [18], [19]. MIP is prepared by copolymerization of a cross-linking agent with the complex formed from a template and polymerizable monomers that have functional groups specifically interacting with the template through covalent or non-covalent bonds. After the template is removed from the resulting polymer matrix, binding sites having the size and shape complimentary to the template are generated. And thus the MIP adsorbent can effectively recognize specific adsorbate from more complex environmental matrices [20], [21]. For example, MIP was used to remove α-estradiol and 17β-estradiol from water samples [22]–[24]. Although the MIP adsorbents have been successfully used to remove many pollutants from water mentioned above, the separation of CA using the MIP adsorbent has hardly been reported. Therefore, using molecularly imprinted polymers as an adsorbent to separate CA from environmental water samples will be considered in the present study.

In this research, we prepared a surface molecularly imprinted polymer using monodispersed DVB homopolymers [poly(DVB)] as a core and CA-imprinted 2-VP–DVB copolymer as a shell by precipitation polymerization. The objective of this study is to prepare and characterize CA imprinted polymers that can be used effectively for removal of CA from environmental water sample. The characteristics of the MIP, including adsorption properties, adsorption selectivity, and the effect of pH and total dissolved solid (TDS) were investigated in detail. Additionally, the resultant MIP was evaluated for the separation of CA from spiked surface water. Finally, the regeneration recognition selectivity of the MIP was also studied.

## Materials and Methods

### Chemicals

Clofibric acid (CA), Carbamazepine (CBZ), divinylbenzene 80(DV8-80), 2-vinylpyridine (2-VP), and 2,2′-azobisisobutyronitrile (AIBN) were all purchased from Sigma-Aldrich ((St.Louis, MO, USA). HPLC grade acetonitrile, methanol, toluene and acetic acid were purchased from Tedia Company, Inc (USA). Ultra–pure water was produced by a Milli-Q water purification system (Millipore, Bedford, MA, USA). AIBN was recrystallized in methanol prior to use.

Standard stock solutions of CA (2 g L^−1^) and CBZ (2 g L^−1^) were prepared in Millipore water and methanol: water (1∶1, v:v) mixture, respectively, and stored at 4°C.

### Apparatus and Analytical Methods

The HPLC analyses were carried out on an Agilent 1200 (Agilent Technologies, USA) HPLC system equipped with a diode array detector (DAD). The UV detection wavelength was 230 nm and the column temperature was set at 30°C. A SHMADZU C_18_ reversed-phase column (250 mm×4.6 mm id, particle size 5 µm) was used for separation. The mobile phase consisted of 60% methanol and 40% Millipore water (0.1% acetic acid). The flow rate was 1.0 mL min^−1^, and the injection volume was 20 µL. Samples were filtered through a 0.45 µm syringe filter (Millipore) before injection and quantification of CA and CBZ was performed using an external standard method. The linear range was established between 0.1 and 1.0 mg L^−1^ with a correlation coefficient (R^2^) of 0.9991. For CA and CBZ, the limit of detection (LOD) was 0.01 mg L^−1^ and the limit of quantitation (LOQ) was 0.1 mg L^−1^.

### Preparation of MIP by Precipitation Polymerization

MIP synthesis involves two successive precipitation polymerization processes. In the first reaction stage, 5.03 mmol of DVB-80 was mixed with 0.085 mmol of AIBN in a 300 mL screw-capped glass bottle followed by addition of 50 mL toluene. Before polymerization, the mixture solution was deoxygenated by purging with nitrogen for 10 min (to remove oxygen) and sealed under nitrogen, then the mixture was polymerized in water-bath shaker (200 rpm) at 65°C for 8 h, resulting in the formation of monodispersed DVB homopolymers [poly(DVB)] used as a core. For the second reaction stage, CA (160.98 mg, 0.75 mmol), 2-VP (0.20 mL, 1.83 mmol), DVB-80 (0.644 mL, 4.52 mmol) and AIBN (13.96 mg, 0.085 mmol) dissolved in 50 mL of acetonitrile/toluene (50∶50, v:v) was introduced to the same reaction bottle and deoxygenated by purging with nitrogen for 10 min and sealed under nitrogen, and then the mixture was polymerized in water-bath shaker (200 rpm) at 65°C for 22 h, resulting in the formation of the imprinted shell. After polymerization, the resultant polymer was Soxhlet extracted with methanol/acetic acid (9∶1, v:v) to remove the template (CA) and filtered. This procedure was repeated several times until CA could not be detected in the filtrate. The remaining polymer was dried under vacuum at 60°C and was used in subsequent experiments. The corresponding non-imprinted polymer (NIP) was prepared in the same manner but in the absence of template. To establish the reproducibility of the MIP preparation protocol, three batches of polymer strictly following the protocol outlined above were conducted.

The specific surface areas of the polymers were measured by nitrogen sorption porosimetry performed on an ASAP 2020 Accelerated Surface Area and Porosimetry Analyzer (Micromeritics Instrument Corporation, Norcross, GA); specific surface areas were calculated using the BET method. Scanning electron micrographs were obtained using a TESCAN TES5136MM Scanning Microscope (Tescan, Chech).

### Adsorption Characteristics of MIP

To investigate the adsorption kinetics of MIP, adsorption efficiency of CA at an initial concentration of 300 mg L^−1^ (5 mL) to the MIP was measured as a function of time. To evaluate the adsorption capacity of the MIP obtained, static adsorption tests were carried out. Ten milligrams of polymer were added to a 10-mL flask containing 5 mL CA solutions of various concentrations (50-1,500 mg L^−1^). After being shaken for 2 h at room temperature, the samples were centrifuged and filtered. The free CA concentration in the filtrate was detected by HPLC. The adsorption of CA to NIP was also measured in a similar manner.

### Selective Recognition Experiments

To investigate the selectivity of the MIP adsorbents, 10 mg of MIP or NIP were added to a 10-mL flask containing a 5 mL mixture of CA (100 mg L^−1^) and CBZ (100 mg L^−1^). After being shaken for 2 h at room temperature, the samples were centrifuged and filtered. The CA and CBZ concentrations in the filtrate were detected by HPLC.

### Effect of pH

The initial water samples pH was adjusted from 3 to 12 with the initial CA concentration at 300 mg L^−1^ by small amounts of HCl or NaOH solution. Then water samples with different pH values were applied to 10 mg MIP or NIP, respectively. After being shaken for 2 h at room temperature, the samples were centrifuged and filtered. The CA concentration in the filtrate was detected by HPLC.

### Selective Separation of CA from Surface Water

Surface water sample collected from the Huangpu River, Shanghai was spiked with 300 mg L^−1^. 10 mg of MIP was added to a 10-mL flask containing 5 mL of spiked surface water sample. After being shaken for 2 h at room temperature, the samples were centrifuged and filtered. The free CA concentration in the filtrate was detected by HPLC.

### Regeneration/reuse of MIP

After CA was adsorbed onto the MIP in spiked lake water for 2 h, the MIP was washed with 5 mL of methanol/acetic acid mixture (9∶1, v:v) and then washed with 5×1 mL of methanol, dried in a vacuum, and reused in the next cycle of sorption experiments.

### Statistical Analysis

One-way ANOVA was performed to assess the significance of differences Statistical significance was evaluated at *p*<0.05 level. All statistical analyses were performed with SPSS software (Ver 13.0; SPSS, Chicago, IL, USA). The experimental data were expressed as mean±standard deviation (SD).

## Results and Discussion

### Characterization of the MIP

**Figure 1:**
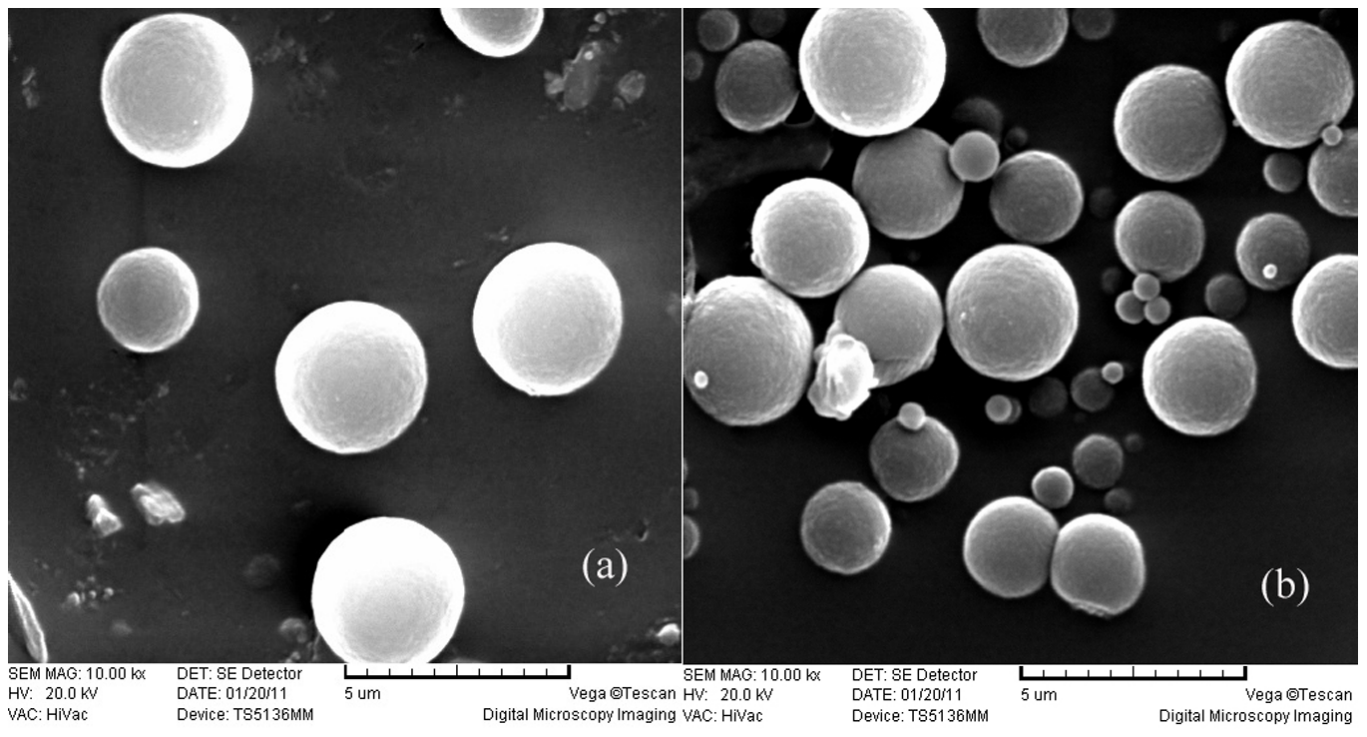
Scanning electron microscopy of the MIP (a) and NIP (b).

The morphology of MIP and NIP were observed by scanning electron microscopy (SEM). As shown in Fig. 1, the well shaped particles had been produced and isolated in the form of discrete polymer with narrow particle size distributions and average diameters (2.9±0.8 µm) in the low micrometre size range. The MIP seems to possess spherical structures with rough surfaces. Spherical molecular imprinting polymers have large surface area, indicating that large number of effective imprinting sites could exist in the surface to rebind the template molecules in aqueous media. Nitrogen sorption porosimetry showed that MIP had well-developed pore structures in the dry state and the specific surface area, pore volume and pore size were 512±7 m^2^ g^−1^, 0.21±0.07 cm^3^ g^−1^ and 2.29±0.4 nm, respectively. For NIP, the specific surface area, pore volume and pore size were 457±5 m^2^ g^−1^, 0.19±0.03 cm^3^ g^−1^ and 2.26±0.6 nm respectively, which were not significantly different from those of MIP (p>0.05). Therefore, the distinct adsorption properties for MIP and NIP could not entirely be attributed to the morphological differences but to the imprinting effect.

### Adsorption Kinetics

**Figure 2:**
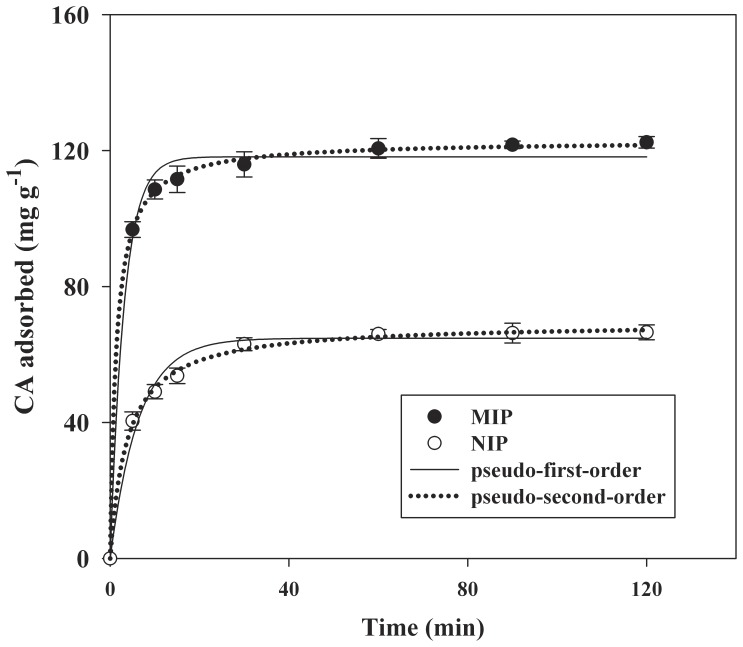
Adsorption kinetics of MIP and NIP for CA (mean ±SD, n = 3).

Fig. 2 shows the adsorption kinetics of CA onto MIP and NIP. It was noted that the adsorption on MIP was as fast as that on NIP,which can be due to no significant morphological difference between MIP and NIP (p>0.05). The adsorption efficiency of CA by MIP reached 122.4±5.6 mg g^−1^ after 2 h adsorption, which was higher than 66.5±2.3 mg g^−1^ of the NIP (p<0.01), suggesting the good imprinting effect of the MIP. As shown in Fig. 2, the adsorption process of MIP could be divided into a quick step and a slow step. In the first step, the adsorption rate was fast, and the contact time to nearly reach equilibrium was 15 min. In the subsequent step, adsorption was slow to reach equilibrium, and the final adsorption efficiency of CA onto MIP reached 82.6±4.1%. As a result, higher adsorption efficiency was realized in a shorter time.

The pseudo-first-order and pseudo-second-order models were applied to describe the sorption kinetic of MIP [25]. They are described in Eqs. (1) and (2).
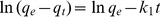
(1)

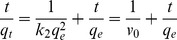
(2)Where q_e_ and q_t_ are the amount of CA adsorbed (mg g^−1^) onto sorbent at the equilibrium and time t (min), respectively. k_1_ is the rate constant of pseudo-first-order adsorption(min^−1^); k_2_ is the rate constant of pseudo-second-order adsorption (g mg^−1^ min^−1^); *v*_0_ represents the initial sorption rate (mg g^−1^ min^−1^).

As shown in Fig. 2 and Table 1, the pseudo-second-order model fitted the experimental data better than the pseudo-first-order model according to the correlation coefficient (r^2^). The good fit (r^2^>0.999) obtained using the second-order model indicated that the sorption of CA onto the MIP and NIP adsorbents conformed to the chemical reaction mechanisms involving valence forces through sharing or exchange of electrons between sorbent and adsorbate.

**Table 1:** Kinetic parameters of the pseudo-first-order and pseudo-second-order equations for CA adsorption onto the MIP and NIP.

Adsorbents	Pseudo-first-order			Pseudo-second-order			
	k_1_ (min^−1^)	q_e_ (mg g^−1^)	R^2^	k_2_ (g mg^−1^ min^−1^)	q_e_ (mg g^−1^)	*v*_0_ (mg g^−1^ min^−1^)	R^2^
MIP	0.0478±0.0005	121.7±7.6	0.935	0.005±0.002	123.5±10.2	76.3±4.1	0.9996
NIP	0.0639±0.0007	66.1±3.9	0.958	0.0041±0.001	68.9±6.8	19.6±1.6	0.9992

### Adsorption Isotherms

**Figure 3:**
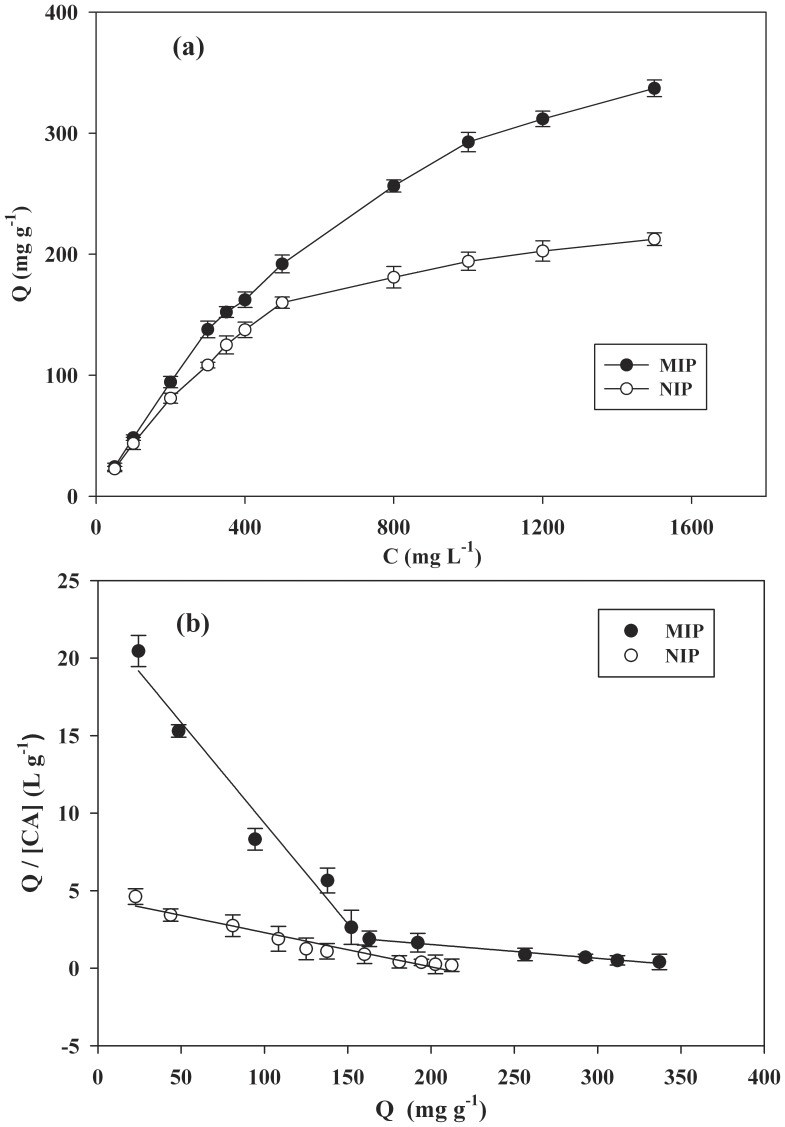
Adsorption isotherms (a) and Scatchard plots (b) of MIP and NIP (mean ±SD, n = 3).

To evaluate the binding affinity of MIP for CA, a saturation adsorption experiment and subsequent Scatchard analysis were conducted. For this purpose, the adsorption isotherms were determined at different initial CA concentrations. As shown in Fig. 3a, the amount of CA bound to the MIP and NIP at adsorption equilibrium increased with the increasing of initial concentration of CA. However, the amount of CA bound to the MIP was higher than that bound to the NIP (p<0.05). This suggested that the imprinted cavities of the MIP may cause the high affinity adsorption of the template to the polymer.

The Scatchard model is always used to assess the binding site heterogeneity of a solid material, such as MIP. The sites can be classified in several families depending on their binding energy beside the template. Each family is characterized by its equilibrium dissociation constant (K_d_, mg L^−1^), as well as by its maximum adsorption capacity (Q_max_, mg g^−1^) [26]. In order to validate the Q_max_ values of the MIP and further to understand its binding characteristic, Scatchard model was used. The Scatchard equation was as follows:
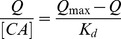
(3)Where Q is the amount of CA bound to CA-MIP at equilibrium, Q_max_ repesents the apparent maximum adsorption capacity, [CA] is the free CA concentration at equilibrium and K_d_ is the dissociation constant. The values of K_d_ and Q_max_ could be calculated from the slope and intercept of the linear curve plotted as Q/[CA] versus Q.

As shown in Fig. 3b, the Scatchard plot for MIP is not a single linear curve, but consists of two linear parts with different slopes. From the slope and intercept of the Scatchard plot, the equilibrium dissociation constant K_d_ and the apparent maximum Q_max_ of the affinity binding sites can be calculated. The linear regression equation for the left part of the curve of MIP is y = −0.133x +22.5 (r = 0.98). K_d_ and Q_max_ can be calculated as 7.52±0.46 mg L^−1^ and 169±5.7 mg g^−1^ of dry polymer. The linear regression equation for the right part of the curve is y = −0.00879x +3.26 (r = 0.99). K_d_ and Q_max_ can be calculated as 114±4.2 mg L^−1^ and 371±7.4 mg g^−1^ of dry polymer. It may be concluded that the binding site configuration in the MIP is heterogeneous in respect to the affinity for CA. The two kinds of binding sites may be produced in the process of polymerization, CA formed two kinds of complexes with 2-VP, and they were fixed in the MIP matrix. After removal of the template, two types of binding sites with distinct affinity existed in the MIP. Similar observations have been reported in the literature [27]. Imprinted polymers synthesized according to the non-covalent approach exhibited heterogeneous binding sites due to incomplete association between the template and the functional monomer. The association is supposed to be mainly responsible for MIP specificity. As a result of that, free functional monomer occurring in the polymer caused non-specific binding sites. Compared to the commercial activated carbon, the MIP in this study has higher binding capacity for sorption of CA than activated carbon sorption reported in the literature [28]. The adsorption of CA to the NIP was also analyzed by Scatchard method. The Scatchard plot of NIP was a single straight line, and the homogeneous binding sites of the NIP had K_d_ and Q_max_ values of 45.2±2.3 mg L^−1^ and 204±8 mg g^−1^, respectively.

### The Adsorption Selectivity of MIP

**Figure 4:**
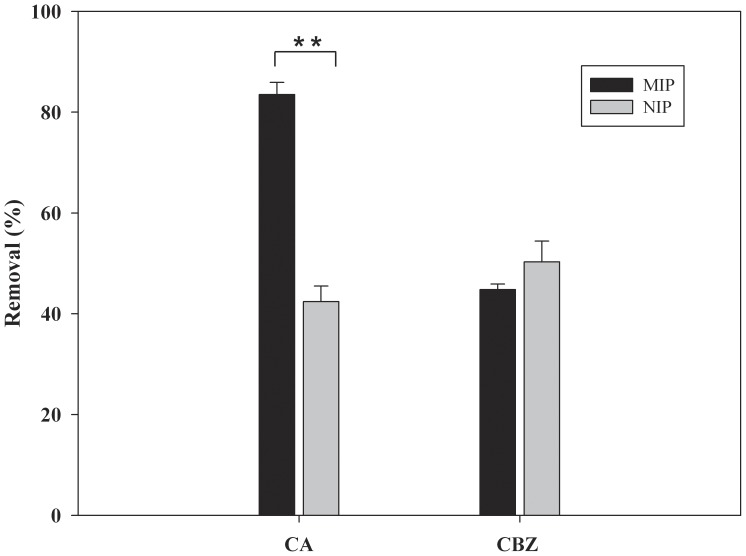
Adsorption selectivity of MIP (mean ±SD, n = 3; *p<0.05).

To evaluate the adsorption selectivity of MIP, CBZ was selected as potential interfering compound. Because the chemical structure of CBZ is similar to that of CA at a certain extent and it also widely coexists with CA in water bodies. As shown in Fig. 4, MIP exhibited good binding affinity for the template molecule. The adsorption efficiency of CA by MIP is significantly higher than that of the structural analog CBZ (p<0.01). The same hydrogen bond may form between the structural analog and the functional monomers due to the similar structure to the template molecule. However, the adsorption efficiency of CBZ by MIP was much lower than for the template molecule. The results showed that the MIP had higher molecular recognition selectivity to its template. The adsorption efficiency of NIP for CA was lower than that of MIP, but their adsorption capacities for the structural analogs were close to each other. These results suggested that the imprinting method created a micro-environment based on the interaction of size, shape, and functionality to the template [29]. There was no proper cavities and recognition site formed in the NIP, so the NIP bind compounds only by non-specific adsorption [30]. As a result, the NIP adsorbed the template molecule much less than the MIP, and there was no significant difference in adsorption efficiency for the interfering compound. So the high adsorption selectivity of MIP provides an effective method to eliminate interferences of other competitive compounds with CA.

### Effect of pH on the Adsorption of CA

**Figure 5:**
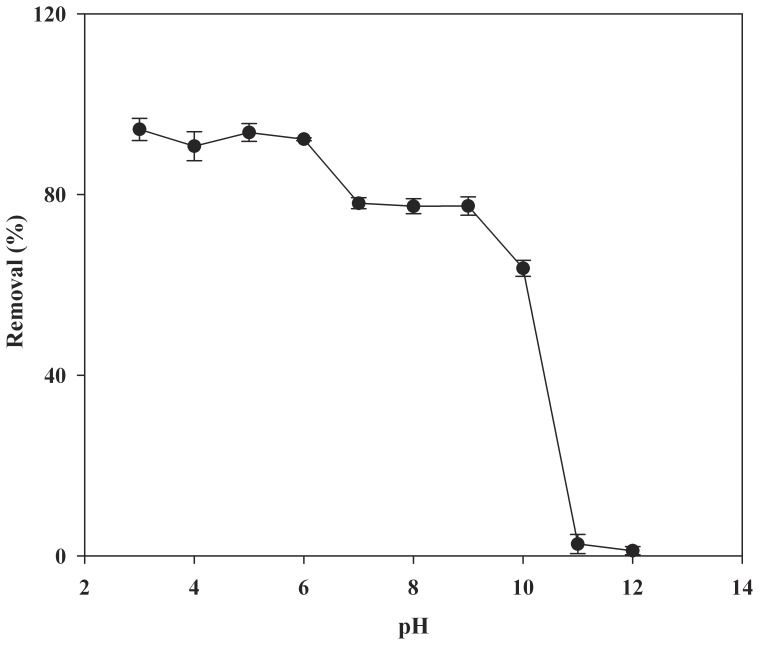
Effect of pH on the adsorption efficiency of CA by MIP (mean ±SD, n = 3).

The pH value of water samples as an important parameter plays a vital role in the adsorption studies. Because pH can influence the dissociation status of target compounds as well as MIP. Therefore, it was necessary to study the effect of pH on adsorption. Fig. 5 shows the effect of pH on the adsorption of CA by MIP in deionized water. As shown in Fig. 5, adsorption efficiency of CA by MIP changed very little when the pH value of the solution was below 6. This suggested that the hydrophobic interaction and binding affinity between CA and the selective binding sites play a predominant role in this pH range. However, the adsorption efficiency of CA decreased significantly with the increase of pH when the pH was between 6 and 12. This phenomenon could be explained by the ionization of CA. The pKa value of CA was 3.18. Ionization would occur for CA under strong basic condition. Therefore, CA was negatively charged. On the other hand, the functional monomer of 2-VP (pKa = 4.98) used in the synthesis of MIP could also be negatively charged. It is known that the –COOH groups in the selective binding cavity of MIP play a key role in the rebinding of target compounds [20]. As a result, the electrostatic repulsive interactions between CA and MIP overcome the binding affinity and hydrophobic interactions became the main driving force during the adsorption at basic pH values. Hence, the adsorption efficiency of CA was reduced. This was consistent with the results obtained by Yu et al. [20]. Almost no CA was adsorbed onto the MIP at initial pH 11 and 12, indicating that the adsorption efficiency of CA by MIP was mainly attributed to the electrostatic attraction and the contribution of other interactions such as hydrophobic interaction and hydrogen binding was extremely limited. The experimental results make it clear that pH 6 was the optimum pH through this study.

### Application to Surface Water: Matrix Effect

**Figure 6:**
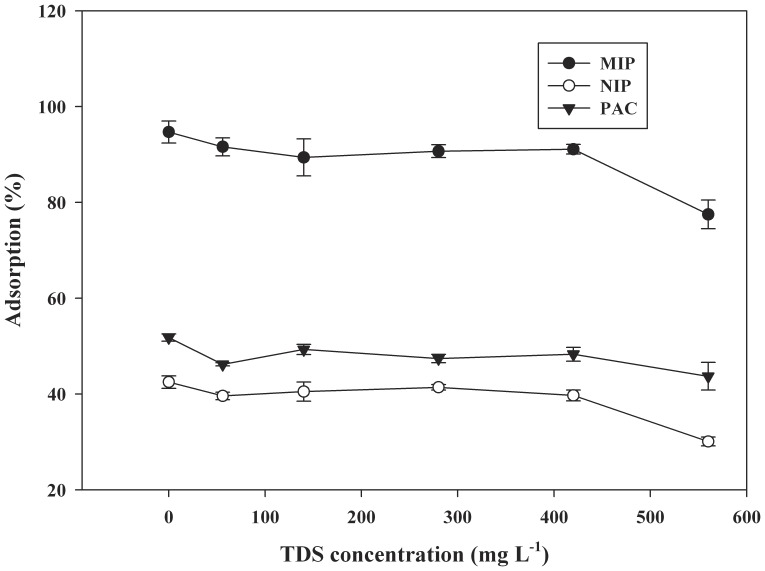
Effect of TDS on the removal efficiency of CA by different adsorbents (mean ±SD, n = 3).

The MIP synthesized was used to remove CA from spiked surface water from the Huangpu River. The adsorption capacity of MIP for CA was compared in the presence of matrix parameters such as dissolved organic matter and total dissolved solids (TDS). The two Q_max_ values for the adsorption of CA from surface water by MIP were 131±4 mg g^−1^ and 359±11 mg g^−1^, which was slightly lower than the adsorption capacity from deionized water (p>0.05). This is expected due to the presence of many different organic and inorganic species in surface water which can also bind onto MIP, hence reducing the effective adsorption of MIP for CA. It is well known that polyvinyl pyridine is efficient ligand for divalent metal ions such as Ca^2+^, Mg^2+^, etc., and can form coordinate bonds with these kinds of metal ions [27]. Therefore the effect of the inorganic ion on the removal of CA by the MIP was studied. The inorganic ions in surface water can be represented by total dissolved solids (TDS) where organic fraction was removed by oxidation of water samples with K_2_Cr_2_O_2_ in acidic conditions at 150°C [31]. The concentration of TDS in Huangpu River was about 560 mg L^−1^, and the chemical oxygen demand (COD) was about 40 mg L^−1^. The interference of inorganic ions was studied by diluting the surface water sample with deionized water and then removal efficiency of CA by MIP was evaluated for CA (Fig. 6). As shown in Fig. 6, when the TDS concentration was about 420 mg L^−1^, there was no significant change in removal efficiency of CA by MIP (p>0.05). This indicated that the adsorption of MIP would be completely retained in the presence of TDS up to 420 mg L^−1^. However, the removal efficiency of CA by MIP decreased as the TDS value exceeds 420 mg L^−1^. This observation may be interfered that the major inorganic ions such as Ca^2+^ and Mg^2+^ could form complexes with functional monomer (2-VP) in the polymer matrix, which may influence the adsorption capacity of MIP. Additionally, it should be emphasized that the removal efficiency of MIP for CA was higher than the uptake by NIP and powdered activated carbon (PAC) under similar conditions (p<0.05). Therefore the MIP specific characteristics were sufficient to remove CA at low concentration from water and wastewater.

### MIP Regeneration/reuse

**Figure 7:**
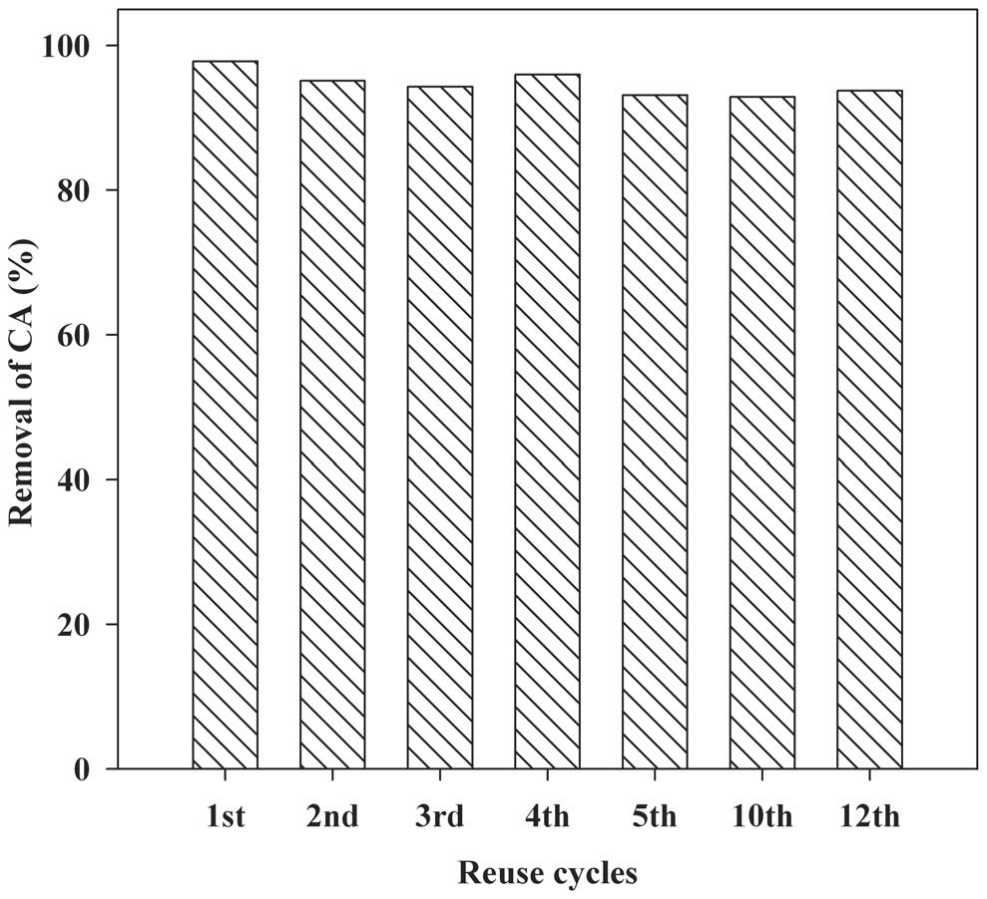
MIP regeneration cycles in spiked lake water.

The regeneration of MIP was investigated in twelve sequential cycles of adsorption–desorption. After adsorption of CA onto the MIP, the MIP was regenerated using the methanol/acetic acid mixture (9∶1, v:v). Fig. 7 shows the adsorption efficiency of the MIP for CA in twelve consecutive adsorption-regeneration cycles. It was shown that the MIP can be used at least 12 cycles without obvious decrease in the adsorption efficiency for CA, which provided evidence that the MIP had certain regeneration adsorption efficiency and could be used repeatedly. Therefore, using MIP as absorbent to remove pollutants from water showed potential to reduce the cost of water treatment.

## Conclusions

A new molecularly imprinted polymer was successfully synthesized with CA as template by precipitation polymerization for the separation of CA from environmental water samples. The results of adsorption experiments indicated that the prepared MIP had high sorption capacity and good selectivity for CA and the sorption of CA was pH dependent. The MIP exhibited excellent adsorption affinity even in the presence of matrix parameters such as TDS in water and MIP can be reused for at least 12 times without loss of performance, indicating the potential application of the MIP adsorbents for selective separation of CA in water or wastewater treatment. Besides water remediation applications, the MIPs developed in this research could be invaluable for the rapid analytical control of CA in environmental pollution monitoring.
